# Fitness and food environments around junior high schools in Taiwan and their association with body composition: Gender differences for recreational, reading, food and beverage exposures

**DOI:** 10.1371/journal.pone.0182517

**Published:** 2017-08-03

**Authors:** Po-Huang Chiang, Lin-Yuan Huang, Meei-Shyuan Lee, Hui-Chen Tsou, Mark L. Wahlqvist

**Affiliations:** 1 Division of Preventive Medicine and Health Services Research, Institute of Population Health Sciences, National Health Research Institutes, Miaoli, Taiwan, ROC; 2 College of Public Health, China Medical University, Taichung, Taiwan, ROC; 3 School of Public Health, National Defense Medical Center, Taipei, Taiwan, ROC; 4 Monash Asia Institute, Monash University, Melbourne, Victoria, Australia; 5 Fuli institute of Food Science and Nutrition, Zhejiang University, Hangzhou City, Zhejiang Province, China; Medical University Innsbruck, AUSTRIA

## Abstract

School environments may contribute to adolescent behavior, reproductive physiology and body composition (BC). The Nutritional and Health Survey in Taiwan (2010) for 1458 junior high school students was geo-mapped for 30 school environs. Facilities for physical activity (fitness centers, gymnasia and sports stadiums, activity centers and parks), sedentary activities (reading material rental shops (RMRS), internet cafes) and food and beverage outlets (FBOs) were calculated as weighted numbers within 1000m of schools. Multiple linear regressions were used to predict BC variable z-scores. For boys, higher fitness center densities and, for girls, gymnasia and sports stadiums were associated with less abdominal fatness. For girls, body mass index, waist circumference (WC) and triceps skinfold thickness (TSF) were greater when RMRS density was higher as was TSF with internet café density. Where there were no FBOs, boys’ WC and TSF were less with more parks, but girls were shorter and WC more adverse. With greater RMRS density and no FBOs, girls still had increased WC/Hip ratio, and less mid-arm muscle circumference. Boys’ findings were more evident after considering puberty. Physical activity facilities (differently by gender), food and beverage outlets absence for boys and low reading material rental shop density for girls increase the likelihood of healthier body composition.

## Introduction

The pubertal transition to adolescence and with continuing growth is one of considerable body compositional change and vulnerability[1]. The changes may have life-long health consequences, albeit on a genetic and epigenetic background, whose trajectory may not be inevitable but, rather, modifiable during this time[2, 3]. Personal behaviors and the home, school and recreational environments are factors to be considered[4–11].

While the increase of childhood obesity is global, occurring even in the face of nutritional deprivation through poor dietary quality[12, 13], the overweight and obesity prevalence in some Asia Pacific regional countries are particularly high, in ascending order (for boys and girls): Japan (23 and 17%), China (24 and 16%), South Korea (25 and 20%), Chile (26 and 27%), Mexico (28 and 29%), the US (30 and 30%) and New Zealand (34 and 34%)[12–14]. In China, overweight (including obesity) prevalence increased over the past 20 years from 14% and 7.4% in 1985 to 34.2% and 30.3% in 2005, for 7-18y males and females, respectively[15]. From 1991 to 2003, the prevalence of adolescent overweight and obesity in Taiwan went from 16.4% to 25.2% in boys and 8.9% to 15.2% in girls[16]. A national cross sectional study in Taiwan between 2006 and 2007 found that the prevalence of overweight and obese adolescents was 19.2% and 10.3% for boys and 12.7% and 3.9% for girls, respectively[17]. The Organization for Economic Co-operation and Development (OECD) report[12] and other evidence from Australia[18] indicates that food insecurity and social disparities with the global financial crisis contributed to increases in obesity prevalence within a short time. However, in France, where there has been active whole-of-community intervention, childhood obesity prevalence has plateaued[12]. Thus, we need to look beyond the simple energy equation of input and output to understand the basis of body compositional disorders affecting fat and lean mass and how to minimize their occurrence. It is known, for example, that emotional health is associated with childhood obesity[19]. For children, especially adolescents, various phenomena may be reflected in personal insecurity, family and personal relationships, educational and sporting achievements, recreational activities and diverse personal behaviors[20, 21]. In an appraisal of support to US food insecure families, girls were more responsive than boys to obesity prevention; gender differences in environmental susceptibility and prevention of body compositional disorders require further elucidation[22]. The gender susceptibility may be more evident at puberty given that being overweight may lead to earlier puberty[23–25]. In Taiwan, the onset of puberty has ranged from 9–14 years of age for boys and 8–13 for girls[26].

It has become clear that, although urbanization does not necessarily pre-date obesity, the built environment may assume characteristics which make it more likely[27]. The school environs are one such setting, negotiated by children in various states of food and other need at least twice a day, alone, with school-mates or with family.

Most attention has been focused on walkability to school and the food outlet exposures which are associated with body composition[7, 28–30]. We have been interested in how other facilities might interact or synergize with food outlets to affect body composition, with implications for later health patterns. In Taiwan, these may include fitness centers and gymnasia, internet and reading material rental shops (for books and magazines directed at adolescents) and public parks. It has been possible to map these across the country and locate them in relation to schools. We hypothesized that these might act co-operatively or detrimentally with food outlets for adolescent body composition. We further hypothesized that the advent of puberty would alter any such associations. To these ends, we have studied Junior High School students and their school environs across Taiwan.

## Materials and methods

### Study participants

Participants were adolescents aged 11–16 years (grade 7–9) who participated in the Nutrition and Health Survey in Taiwan (NAHSIT) 2010–2011. All of Taiwan’s 358 townships/districts were classified into 5 strata (northern 1, northern 2, central, southern, and eastern area) by geographical location and the population density. The survey used the PPS (probabilities proportional to sizes) sampling method to select 1620 students from 30 junior high schools (6 schools from each stratum) randomly[31]. Their demographic and anthropometric information was obtained through interviews and health examinations. The present study excluded participants without body composition information, leaving 1458 junior high school students eligible for analysis. The study was approved by the Institutional Review Board of the National Health Research Institutes, Taiwan.

### Instruments

***The NAHSIT 2010–2011*** was funded by the Department of Health to provide assessment of health and nutrition of junior high school students in Taiwan. It was a cross-sectional and nationally representative survey. The questionnaire-based interviews and anthropometry measurements were conducted by trained personnel. The survey was conducted face-to-face at school with students and their teachers and through household interviews to obtain information regarding family members, socio-demographics, nutritional attitudes and behaviors, physical activity, and diet. Physical examinations were also conducted at school to provide body compositional information and allow venesection for fasting blood samples. All personal information was obtained through the survey with informed consent of parents or guardians.

**Fig 1:**
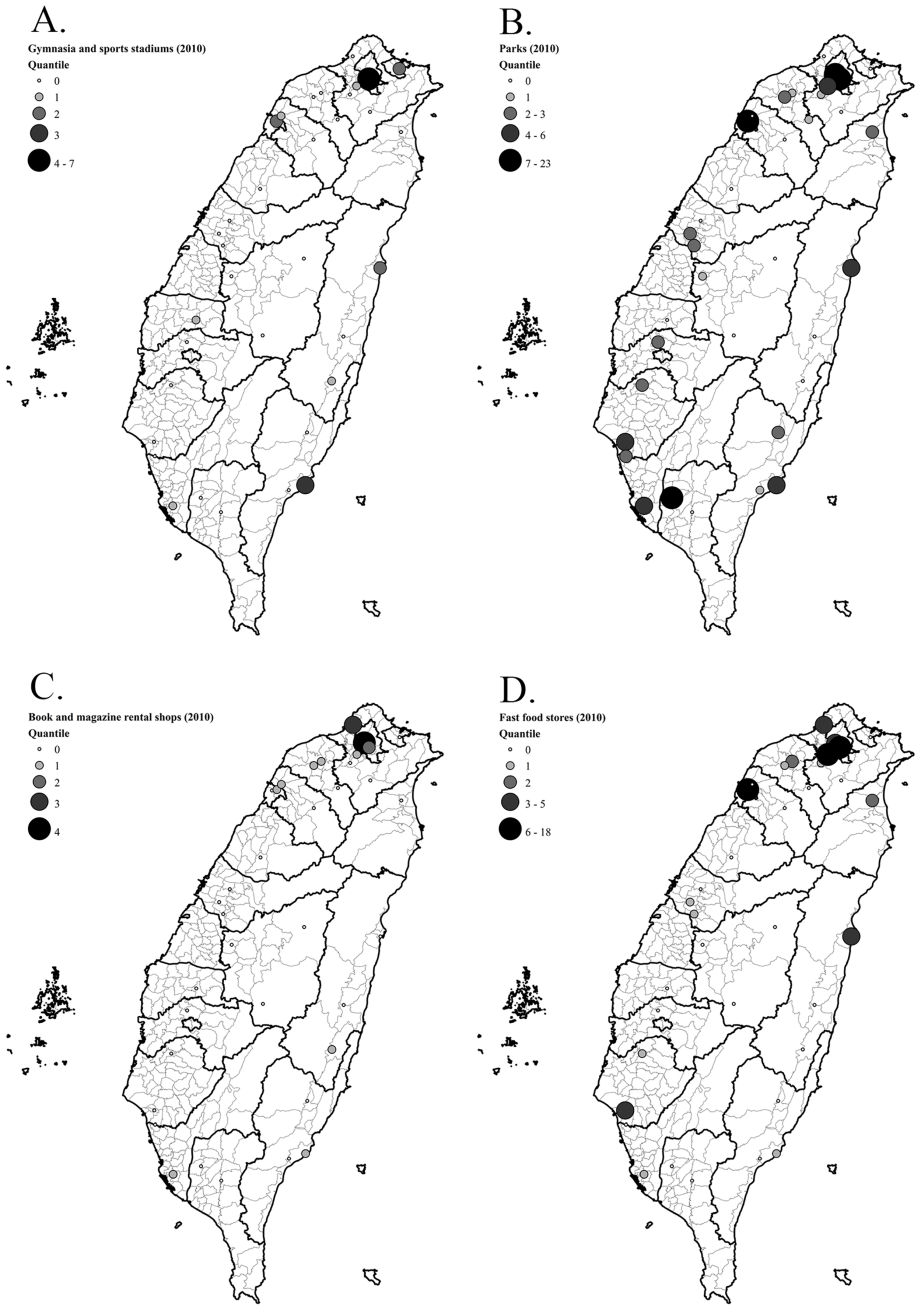
The number of facilities within 1000m around each Taiwanese junior high school in NAHSIT (2010). (A) gymnasia and sports stadiums; (B) parks; (C) book and magazine rental shops; (D) fast food stores.

***The point of interest (POI) dataset*** for specific point locations was purchased from RITI Technology Inc., and corresponded to the NAHSIT 2010–2011 time frame. We gathered geographic points for food, physical or recreational and sedentary locations in the navigation system and added this layer onto the school environment data sets (Fig 1).

### Procedure

#### Geocoded mapping and buffer analysis

Junior high school addresses were obtained from the NAHSIT data and transferred to a geocoded database with Geo Gadget designed by the Center for Geographic Information Science (GIS), Academia Sinica, Taiwan. We applied Geo Gadget to transfer 30 schools’ geocoded nominal data into spatial coding with XY coordination for analysis. Obesogenic environments around the school were classified into 3 groups, (1) those to do with food (convenience, fast food, and beverage shops), (2) physical or recreational activity (fitness centers, gymnasia and sports stadiums, activity centers, and parks), and (3) sedentary activity (book and magazine rental shops and internet cafes). All locations of food store and activity facilities information were part of the POI datasets. Convenience stores included 7–11, Family Mart, OK, Hi-Life and Niko Mart. Fast food stores included McDonald’s, KFC and Burger King. Beverage stores were identified as providers of sweet beverages. Fitness centers were identified as a place of business with equipment and facilities for exercising and improving physical fitness. Gymnasia and sports stadiums were identified as places designed and equipped for indoor or outdoor sports, exercise, or physical education. Activity Centers were defined as a place that attracts people for dancing, working, studying, recreation or socializing. Parks can be used for pleasure and exercise. People can rent magazines, novels (e.g. classic, historical, mystery, romance and science-fiction), and comic books and so on in book and magazine rental shops. The internet cafe is a place which provides internet access to the public, usually for a fee, and these businesses usually provide snacks and drinks.

A circular buffer of 1000 meters was calculated around each school by ArcGIS (ArcInfo, version 10.2; ESRI Inc., Redlands, CA, USA), corresponding to an approximate 12–15 minutes’ walk distance at 4–5 km/hour[32–34]. Food store and activity facility availabilities were computed within this buffer area. For a measure of accessibility of food store and physical activity facilities, the weighted number of facilities was calculated and summed according to their distances from each school, with a weight of 1 for those with distances smaller than 200 m, 0.75 for those between 200–500 m, and 0.5 for those between 500 m and 1000 m[35].

#### Demographics

Covariates considered in the present study included age, father’s ethnicity (Fukienese, Hakka, Mainlander, and Indigenous), mother’s education (up to primary school, secondary education, and above), household income (0–30,000, 30,000–50,000, 50,000–80,000, >80,000 NTD/month), ever smoking (no, yes), drinking alcohol (no, yes), dietary quality (the Youth Healthy Eating Index-Taiwan; YHEI-TW), physical activity (moderate or heavy physical activity ≥ 30 min/d), read on weekdays (0–1, 1–3, ≥3 hr/d), and development of puberty (boys: beard growth (not yet, at first, in progress, completed); girls: menarche (yes, no)).

***Personal behaviors***. Habits like reading, watching TV, playing computer games, were recorded.

#### Physical measurements

**Fig 2:**
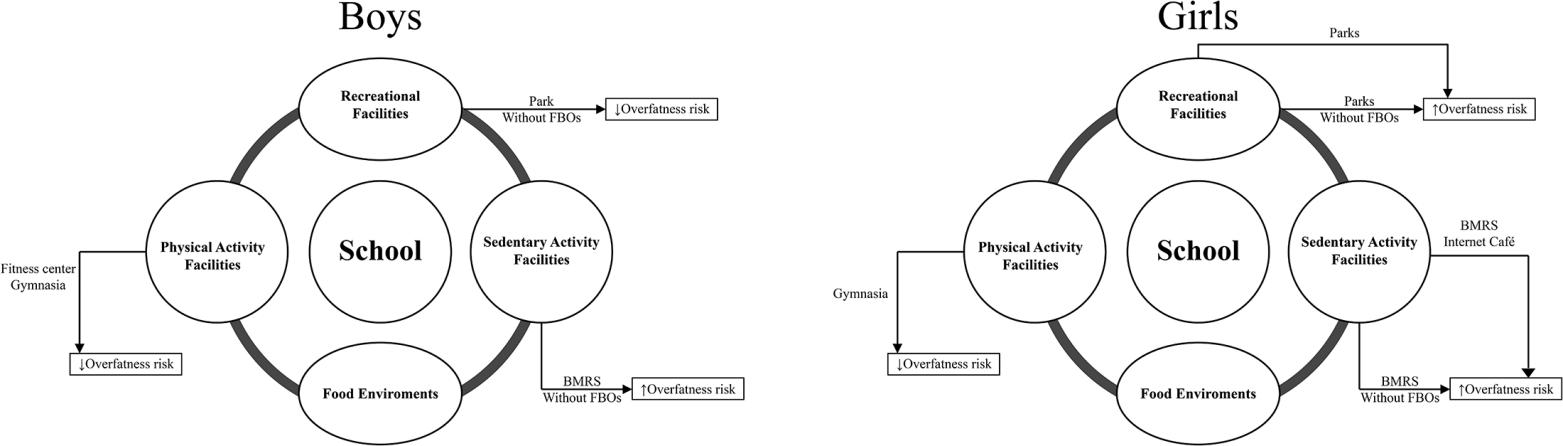
School environmental risk factors for overfatness in boys and girls based on adjusted multivariable regression analyses. “Overfatness” is used to indicate any of the following: (a) BMI (body mass index), (b) TSF (triceps skinfold thickness), and (c) WC (waist circumference). RMRS, reading material rental shops; FBOs, food and beverage outlets.

***Anthropometrics*** including height, weight, body mass index (BMI), waist circumference (WC) and triceps skinfold thickness (TSF) were collected during the NAHSIT physical examinations conducted at the schools. Muscle mass was determined by anthropometric measurement of the mid upper arm where circumference and TSF allowed its computation as Mid Arm Muscle Circumference (MAMC = MAC–(π*TSF)). We also calculated the ratio of WC/Height and WC/Hip. Adolescents were in the pubertal stage of physical development. Adolescents’ growth rates differ greatly in this age group, and therefore we calculated gender-age-specific z-scores for height, weight, BMI, WC, TSF, and MAMC. For purposes of classification of weight disorders, we used the Taiwanese Growth Charts (2002 year) where, for age and gender, underweight and overweight were BMI centiles <15% or ≥ 85%, respectively, and obesity was a BMI centile ≥ 95%. By these criteria, we defined “body compositional disorders (BCD)” such as underweight, overweight and overfatness, the latter taking into account BMI, WC, and TSF with lean mass indicator by MAMC. Evidence that a putative environmental risk factor is associated with BMI, WC and TSF, as the dependent variables, on multivariable regression analysis, was provided by the significance of the β coefficient (Fig 2). ***Blood pressure*** (systolic blood pressure (SBP) and diastolic blood pressure (DBP)) was taken.

#### Blood analytes

Fasting blood was obtained by venesection for plasma metabolic analytes (glucose, total cholesterol, triglycerides, HDL, LDL, and uric acid).

### Data analysis

Statistical analyses were performed using SAS software version 9.1 for Windows, and SUDAAN (Survey Data Analysis)[36]. SUDAAN was used to adjust for the design effects of cluster sampling and to obtain unbiased estimates of standard errors. The distributions of basic characteristics among adolescents according to gender were assessed by chi-square and t tests. Multiple linear regression analysis was used to compare schoolchildren’s body composition and the number of food outlets among regions. Models were adjusted for father's ethnicity, mother’s education, household income, smoking, drinking alcohol, dietary quality (YHEI-TW), physical activity (moderate or heavy physical activity ≥ 30 min/d), reading during weekdays and development of puberty (boys: beard growth; girls: menarche). Statistical significance was defined as *P*<0.05.

## Results

The characteristics of junior high school students by gender are shown in Tables 1 and 2. Their mean age was 13.5 years and ranged from 11 to 16 years. Socio-economic information about parents and students is provided. In particular, the prevalence of post-secondary education for both mothers and fathers was high at around or above 60%, which may represent a selective advantage for their children in high school attendance, since these education profiles exceed the national averages (40%) [37].

**Table 1:** Demographic characteristics of junior high school students by gender (N = 1458, 11–16 years old).

Characteristics	Overall	Boys	Girls	*p* value^a^
	Mean (SE) or N (%)			
Participants (%)	1458 (100%)	703 (52.0%)	755 (48.0%)	
Age (years)	13.5 (0.07)	13.5 (0.08)	13.5 (0.09)	0.974
Father’s ethnicity				0.164
Fukienese	974 (75.8%)	474 (75.0%)	500 (76.7%)	
Hakka	228 (13.7%)	116 (14.6%)	112 (12.6%)	
Mainlander	147 (8.43%)	66 (7.62%))	81 (9.32%)	
Indigenes	80 (2.14%)	36 (2.76%)	44 (1.45%)	
Father’s educational level				0.028
Primary and below	47 (3.48%)	22 (3.09%)	25 (3.90%)	
Secondary education	495 (37.7%)	219 (33.3%)	276 (42.6%)	
University and above	805 (58.8%)	402 (63.7%)	403 (53.5%)	
Mother’s educational level				0.078
Primary and below	59 (3.44%)	28 (3.60%)	31 (3.27%)	
Secondary education	442 (31.3%)	186 (27.5%)	256 (35.5%)	
University and above	848 (65.3%)	432 (68.9%)	416 (61.3%)	
Household income				0.402
0–30,000 NTD/month	295 (19.5%)	129 (18.1%)	166 (21.0%)	
30,000–50,000 NTD/month	289 (22.6%)	137 (22.0%)	152 (23.3%)	
50,000–80,000 NTD/month	369 (29.7%)	195 (32.4%)	174 (26.7%)	
> 80,000 NTD/month	353 (28.3%)	163 (27.6%)	190 (29.1%)	
Ever smoking				0.012
No	800 (90.0%)	383 (86.6%)	417 (93.8%)	
Yes	91 (10.0%)	59 (13.4%)	32 (6.16%)	
Drinking alcohol				0.371
No	792 (87.7%)	400 (89.2%)	392 (86.1%)	
Yes	98 (12.3%)	42 (10.8%)	56 (13.9%)	
Read during weekdays				0.961
0-1hrs/day	393 (46.4%)	201 (46.8%)	192 (45.9%)	
1–3 hrs/day	339 (41.6%)	164 (41.6%)	175 (41.7%)	
≥3 hrs/day	105 (12.0%)	49 (11.6%)	56 (12.5%)	
Watch TV during weekdays				0.316
0-1hrs/day	454 (57.3%)	220 (55.0%)	234 (59.9%)	
1–3 hrs/day	301 (34.4%)	163 (37.8%)	138 (30.6%)	
≥3 hrs/day	82 (8.26%)	31 (7.17%)	51 (9.52%)	
Play computer games during weekdays				0.032
0–1	525 (62.0%)	242 (57.1%)	283 (67.6%)	
1–3 hrs/day	238 (29.9%)	130 (32.6%)	108 (26.7%)	
≥3 hrs/day	73 (8.13%)	41 (10.2%)	32 (5.69%)	
Moderate or heavy physical activity				<0.001
≥ 30 min/day	208 (25.5%)	144 (35.9%)	64 (13.4%)	
< 30 min/day	626 (74.5%)	269 (64.1%)	357 (86.6%)	
Dietary score (YHEI-TW)	48.1 (0.47)	48.2 (0.61)	48.1 (0.56)	0.937
Food consumption frequency (times/week)				
Fast foods from consumption	0.45 (0.04)	0.41 (0.05)	0.49 (0.04)	0.116
Sugary beverages consumption	5.33 (0.17)	5.63 (0.27)	5.01 (0.26)	0.139
Dairy products consumption				
Milk	3.53 (0.18)	3.79 (0.26)	3.25 (0.22)	0.098
Flavor milk	0.73 (0.07)	0.75 (0.10)	0.72 (0.06)	0.763
Yoghourt	0.48 (0.06)	0.46 (0.08)	0.51 (0.06)	0.574
Cheese	0.86 (0.08)	0.80 (0.11)	0.93 (0.10)	0.407

^a^ Test of the distributions across gender by t-tests and chi-square tests.

**Table 2:** Puberty and anthropometric characteristics of junior high school students by gender (N = 1458, 11–16 years old).

Characteristics	Overall	Boys	Girls	*p* value^a^
	Mean (SE) or N (%)			
Development of puberty				
Menarche (girls only)				
Yes			413 (93.8%)	
No			32 (6.23%)	
Development of breast (girls only)				
Not yet			8 (27.6%)	
At first			8 (30.3%)	
In progress			12 (42.0%)	
Development of height				<0.001
Not yet	30 (3.70%)	25 (6.22%)	5 (0.82%)	
At first	128 (13.7%)	93 (19.4%)	35 (7.24%)	
In progress	626 (72.2%)	300 (72.1%)	326 (72.2%)	
Completed	73 (10.5%)	9 (2.26%)	64 (19.8%)	
Development of armpit/pubic hair				<0.001
Not yet	86 (10.1%)	64 (14.7%)	22 (4.86%)	
At first	209 (22.0%)	117 (23.0%)	92 (21.0%)	
In progress	527 (63.5%)	231 (59.3%)	296 (68.2%)	
completed	39 (4.42%)	14 (3.04%)	25 (5.97%)	
Trouble of acne or pimple				0.001
Not yet	95 (11.6%)	63 (15.1%)	32 (7.68%)	
At first	181 (18.6%)	94 (20.4%)	87 (16.6%)	
In progress	544 (64.1%)	242 (58.3%)	302 (70.5%)	
Completed	11 (1.17%)	3 (1.04%)	8 (1.32%)	
No trouble	35 (4.54%)	21 (5.12%)	14 (3.91%)	
Change of voice (boys only)				
Not yet		104 (23.3%)		
At first		106 (21.4%)		
In progress		172 (43.9%)		
Completed		38 (11.4%)		
Grow beards (boys only)				
Not yet		146 (33.9%)		
At first		146 (31.7%)		
In progress		125 (32.9%)		
Completed		8 (1.43%)		
Body composition				
Height (cm)	161 (0.25)	164 (0.35)	157 (0.22)	<0.001
Weight (kg)	54.2 (0.48)	57.5 (0.67)	50.5 (0.47)	<0.001
Body mass index (BMI, kg/m^2^)	20.8 (0.15)	21.1 (0.22)	20.5 (0.18)	0.027
BMI categories				0.002
%Underweight	193 (13.1%)	103 (14.7%)	90 (11.4%)	
%Normal weight	872 (60.6%)	380 (54.2%)	492 (67.4%)	
%Overweight/Obesity	393 (26.3%)	220 (31.1%)	173 (21.2%)	
BMI categories in relation to puberty^b^				
% Underweight before onset		32 (20.1%)	11 (33.4%)	
% Underweight after onset		40 (13.9%)	41 (7.78%)	
% Overweight/Obesity before onset		43 (30.2%)	7 (21.4%)	
% Overweight/Obesity after onset		89 (32.4%)	93 (20.8%)	
Triceps skin fold thickness (TSF, mm)	16.6 (0.43)	14.2 (0.52)	19.3 (0.47)	<0.001
Mid Arm Muscle Circumference (MAMC, cm)	20.0 (0.14)	21.4 (0.17)	18.4 (0.16)	<0.001
Waist circumference (WC, cm)	74.2 (0.44)	75.0 (0.58)	73.4 (0.51)	0.019
WC/Height ratio	0.46 (0.001)	0.46 (0.001)	0.47 (0.001)	0.018
WC/Hip ratio	0.82 (0.001)	0.83 (0.001)	0.81 (0.001)	<0.001
Blood pressure (mmHg)				
Systolic blood pressure (SBP)	105 (0.55)	109 (0.66)	100 (0.50)	<0.001
Diastolic blood pressure (DBP)	60 (0.66)	60 (0.67)	60 (0.75)	0.441
Plasma metabolic analytics				
Fasting glucose (mg/dL)	95.4 (0.40)	96.2 (0.48)	94.4 (0.57)	0.012
Total cholesterol (mg/dL)	158 (1.37)	156 (1.70)	161 (1.90)	0.055
Triglycerides (mg/dL)	71.8 (1.75)	72.3 (2.93)	71.2 (1.78)	0.749
HDL (mg/dL)	55.1 (0.66)	53.8 (0.82)	56.7 (0.78)	0.004
LDL (mg/dL)	88.7 (1.16)	87.6 (1.50)	90.0 (1.58)	0.250
Uric acid (mg/dL)	5.76 (0.06)	6.53 (0.08)	4.89 (0.06)	<0.001

^a^ Test of the distributions across gender by t-tests and chi-square tests.

^b^Onset of puberty in boys was identified by beards growth, in girls was menarche.

For potentially adverse *personal behaviors*, smoking was admitted by 10% of students (13% for boys and 6% for girls) and drinking alcohol by 12% (11% for boys and 14% for girls).

Time spent per day on *physical and sedentary activities* (reading, watching TV, and playing computer games) are also shown. *Dietary patterns* are described by way of the YHEI-TW score and in regard to consumption frequency (times/week) of convenience foods, sugary beverages and dairy products. YHEI-TW scores of 0 to 90 are regarded as acceptable, with higher scores indicating better dietary quality [7]. It should be noted that the average use of fast foods in this age group across Taiwan is relatively low at less than once in 2 weeks. By contrast, the use of sugary beverages exceeds 5 times per week and that of liquid and flavored milk combined more than 4 times per week. This amounts to some 2 energy-replete beverages per school-day, if they were consumed on week-days.

In regard to *pubertal development*, we regarded the presence of beard hair as the onset in boys (32% with first indication, 33% in progress and 1% complete); and for girls, the menarche (94% had commenced menstruation).

Indices of *body composition* derived by anthropometry included BMI (kg/m^2^) (a mean of 21.1 for boys and 20.5 for girls); waist circumference (WC in cm) (a mean of 75.0 for boys and 73.4 for girls); mid arm muscle circumference (MAMC in cm, 21.4 for boys and 18.4 for girls); triceps skin fold thickness (TSF in cm) (14.2 for boys and 19.3 for girls);and WC/hip ratio (0.83 for boys and 0.81 for girls).

From BMI it can be seen that 20.1% of boys and 33.4% of girls were underweight and 30.2% of boys and 21.4% of girls overweight before the onset of puberty; after its onset, the respective prevalences, for underweight, were 13.9% for boys and 7.78% for girls and, for overweight, 32.4% for boys and 20.8% for girls.

*Cardiovascular risk factors* are shown by way of systolic and diastolic blood pressure (BP) (mean of 105/60) and metabolic variables (fasting plasma glucose (mg/dL) (mean of 95.4) and plasma lipids (mg/dL) (means for total cholesterol, triglycerides and HDL cholesterol were 158, 72 and 55 respectively).

*Maps to show the representative junior high school environs* facility densities studied are depicted by dot size in Fig 1. Gymnasia and sports stadiums, parks, book and magazine rental shops and fast food stores are shown in separate panels. It can be seen that the spectrum of densities differs markedly between major cities, provincial towns and coastal, rural or mountainous (Taiwan has a central mountain range) areas and also between the north-west (where Taipei and Taoyuan are situated) and the rest of the country.

There were no significant associations between food environments around junior high schools and student’s body compositions (Table 3).

**Table 3:** Multiple linear regressions for body composition in relation to the food environments within 1000m^a^ around junior high school by gender.

Body composition	Convenience Stores			Fast food stores			Beverage shops		
	β value			β value			β value		
	Model 1	Model 2	Model 3	Model 1	Model 2	Model 3	Model 1	Model 2	Model 3
**Boys**									
Height z-score	-0.007	-0.010	-0.002	-0.008	-0.013	-0.003	-0.018	-0.020	-0.021
Weight z-score	-0.011	-0.011	-0.007	-0.015	-0.017	-0.012	-0.004	-0.004	-0.001
BMI z-score	-0.007	-0.006	-0.005	-0.010	-0.010	-0.009	0.010	0.010	0.014
WC z-score	-0.013	-0.013	-0.012	-0.019	-0.020	-0.020	-0.007	-0.008	-0.006
WC/Height Ratio	-0.001	-0.001	-0.001	-0.002	-0.001	-0.002	-0.001	-0.001	-0.001
WC/Hip Ratio	-0.001	-0.001	-0.001	-0.002	-0.002	-0.002	-0.001	-0.001	-0.001
TSF z-score	-0.013	-0.011	-0.014	-0.017	-0.016	-0.020	-0.018	-0.015	-0.015
MAMC z-score	-0.010	-0.011	-0.003	-0.010	-0.011	-0.0002	0.010	0.009	0.015
**Girls**									
Height z-score	0.015	0.011	0.007	0.016	0.011	0.007	-0.0002	-0.004	-0.010
Weight z-score	0.004	0.006	0.003	0.012	0.013	0.010	0.017	0.021	0.017
BMI z-score	0.0001	0.004	0.001	0.007	0.012	0.009	0.023	0.029	0.026
WC z-score	-0.002	-0.0001	-0.003	-0.002	0.0004	-0.003	0.016	0.019	0.016
WC/Height Ratio	-0.0002	-0.0001	-0.0001	-0.0002	0.0001	-0.0001	0.001	0.002	0.001
WC/Hip Ratio	-0.0002	-0.0001	-0.0001	-0.001	-0.0003	-0.0004	0.0003	0.001	0.001
TSF z-score	0.010	0.013	0.011	0.019	0.022	0.020	0.028	0.030	0.028
MAMC z-score	-0.018	-0.017	-0.019	-0.022	-0.021	-0.023	-0.001	0.0003	-0.002

^a^The weighted numbers of food stores were calculated according to their distances from each school, with a weight of 1 for those with distances smaller than 200 m, 0.75 for those between 200–500 m, and 0.5 for those between 500 m and 1000 m.

BMI, body mass index; WC, waist circumference; TSF, Triceps skinfold thickness; MAMC, Mid Arm Muscle Circumference.

Model 1: adjusted for father's ethnicity, mother’s education, household income, smoking and drinking alcohol.

Model 2: Model 1 + dietary quality (YHEI-TW), physical activity (moderate or heavy physical activity ≥ 30 min/day) and read during weekdays.

Model 3: Model 2 + development of puberty (boys: grow beards; girls: Menarche)

The *models for physical activity and sedentary activity* facilities are shown in Tables 4 and 5. More favorable body composition is seen for boys with greater *fitness center* and also gymnasia and sports stadium density, but, for girls, only with *gymnasia and sport stadium density*. However, this applies to fat distribution as judged by waist circumference (WC) with a significantly negative β coefficient, and not necessarily fat mass with a non-significant β coefficient for BMI. Nevertheless, for girls, while WC/hip ratio is less with the greater availability of gymnasia, so also is muscle mass, judged by the MAMC z-score.

**Table 4:** Multiple linear regressions for body composition in relation to the physical activity facilities within 1000m^a^ around junior high school by gender.

Body composition	Physical activity facilities								
	Fitness centers			Gymnasia and sports stadiums			Activity centers		
	β value			β value			β value		
	Model 1	Model 2	Model 3	Model 1	Model 2	Model 3	Model 1	Model 2	Model 3
Boys									
Height z-score	0.005	-0.007	0.028	-0.010	-0.016	0.020	0.083	0.073	0.073
Weight z-score	-0.060	-0.067	-0.054	-0.042	-0.045	-0.035	-0.015	-0.020	-0.027
BMI z-score	-0.061	-0.064	-0.064	-0.037	-0.039	-0.040	-0.032	-0.033	-0.041
WC z-score	-0.076*	-0.079*	-0.082	-0.052	-0.055	-0.058	-0.038	-0.039	-0.048
WC/Height Ratio	-0.006	-0.006	-0.008	-0.004	-0.004	-0.005	-0.005	-0.004	-0.005
WC/Hip Ratio	-0.007	-0.007*	-0.009*	-0.006**	-0.006**	-0.008***	-0.004	-0.004	-0.005
TSF z-score	-0.056	-0.058	-0.073	-0.024	-0.024	-0.036	-0.036	-0.031	-0.044
MAMC z-score	-0.048	-0.051	-0.015	-0.015	-0.016	0.016	-0.021	-0.028	-0.019
Girls									
Height z-score	0.026	0.010	0.004	0.021	0.013	0.008	0.002	-0.005	-0.004
Weight z-score	0.029	0.035	0.030	0.014	0.016	0.012	-0.004	-0.0002	-0.0004
BMI z-score	0.023	0.037	0.034	0.007	0.013	0.010	0.004	0.012	0.012
WC z-score	-0.036	-0.024	-0.028	-0.056	-0.050	-0.054	0.007	0.008	0.008
WC/Height Ratio	-0.002	-0.001	-0.001	-0.003	-0.003	-0.003	0.001	0.001	0.001
WC/Hip Ratio	-0.006	-0.005	-0.005	-0.006*	-0.006*	-0.006*	-0.001	-0.001	-0.001
TSF z-score	0.046	0.058	0.056	0.029	0.036	0.033	0.030	0.034	0.034
MAMC z-score	-0.071	-0.065	-0.067	-0.061*	-0.058*	-0.061*	-0.029	-0.026	-0.027

^a^The weighted number of facilities was calculated and summation according to their distances from each school, with a weight of 1 for those with distances smaller than 200 m, 0.75 for those between 200–500 m, and 0.5 for those between 500 m and 1000 m.

BMI, body mass index; WC, waist circumference; TSF, Triceps skinfold thickness; MAMC, Mid Arm Muscle Circumference.

Model 1: adjusted for father's ethnicity, mother’s education, household income, smoking and drinking alcohol.

Model 2: Model 1 + dietary quality (YHEI-TW), physical activity (moderate or heavy physical activity ≥ 30 min/day) and read during weekdays

Model 3: Model 2 + development of puberty (boys: grown beard; girls: menarche)

*p<0.05;

**p<0.01;

***p<0.001

**Table 5:** Multiple linear regressions for body composition in relation to the recreational or sedentary activity facilities within 1000m^a^ around junior high school by gender.

Body composition	Recreational facilities			Sedentary activity facilities					
	Parks			Book and magazine rental shops			Internet Cafes		
	β value			β value			β value		
	Model 1	Model 2	Model 3	Model 1	Model 2	Model 3	Model 1	Model 2	Model 3
Boys									
Height z-score	0.003	-0.002	0.002	-0.068	-0.094	-0.096	-0.101	-0.110	-0.087
Weight z-score	-0.001	-0.004	-0.002	0.011	-0.006	-0.013	-0.036	-0.036	-0.023
BMI z-score	-0.002	-0.003	-0.003	0.048	0.041	0.033	0.022	0.025	0.030
WC z-score	0.002	0.001	0.001	0.031	0.022	0.013	-0.043	-0.045	-0.047
WC/Height Ratio	0.0002	0.0003	0.0002	0.004	0.004	0.002	-0.004	-0.004	-0.005
WC/Hip Ratio	-0.0001	0.0001	-0.0001	0.003	0.004	0.003	-0.004	-0.005	-0.006
TSF z-score	0.001	0.001	-0.001	0.0003	0.004	-0.006	-0.066	-0.053	-0.067
MAMC z-score	-0.007	-0.009	-0.004	0.048	0.041	0.042	0.001	-0.006	0.028
Girls									
Height z-score	-0.018	-0.021	-0.025*	-0.085	-0.092	-0.124*	-0.020	-0.017	-0.037
Weight z-score	0.018	0.017	0.015	0.081	0.081	0.056	0.037	0.045	0.030
BMI z-score	0.026*	0.026*	0.024*	0.112*	0.111*	0.092*	0.056	0.065	0.053
WC z-score	0.021	0.020	0.018	0.177**	0.168**	0.148**	-0.013	-0.010	-0.024
WC/Height Ratio	0.002	0.002	0.001	0.012***	0.012***	0.011***	0.001	0.001	-0.0001
WC/Hip Ratio	0.002	0.002	0.001	0.015***	0.014**	0.014**	-0.002	-0.002	-0.002
TSF z-score	0.025*	0.027**	0.025*	0.133*	0.132*	0.117*	0.110*	0.106	0.096
MAMC z-score	-0.001	-0.001	-0.003	0.055	0.051	0.037	-0.071	-0.075	-0.084

^a^The weighted number of facilities was calculated and summation according to their distances from each school, with a weight of 1 for those with distances smaller than 200 m, 0.75 for those between 200–500 m, and 0.5 for those between 500 m and 1000 m.

BMI, body mass index; WC, waist circumference; TSF, Triceps skinfold thickness; MAMC, Mid Arm Muscle Circumference.

Model 1: adjusted for father's ethnicity, mother’s education, household income, smoking and drinking alcohol.

Model 2: Model 1 + dietary quality (YHEI-TW), physical activity (moderate or heavy physical activity ≥ 30 min/day) and read during weekdays

Model 3: Model 2 + development of puberty (boys: grown beard; girls: menarche)

*p<0.05;

**p<0.01;

***p<0.001

With more *parks* in the neighborhoods of the high schools, girls were more likely to have higher BMIs, even when extensive adjustments were made to the models. However, the significance of this association was lost with adjustment for the advent of puberty. This indicates that if park availability is a risk for overfatness in girls, that this might depend on the stage of physiological development.

The greater availability of *book and magazine rental shops* is associated with greater total, central and limb fat in girls, but not in boys. This applies in models which adjust for basic demography, personal behaviors, and reading itself, but not with adjustment for puberty. This suggests that *puberty* is a contributing factor to the risk for overfatness in this setting.

In view of a likely role of puberty in the associations between park and reading material rental shop availabilities, we modeled these in their own right, which is to say, there were 15 schools where no food outlet stores (convenience or fast food) or beverage shops were present in the school neighborhood, as shown in Table 6.

**Table 6:** Multiple linear regression for the body composition in relation to parks and book and magazine rental shops within 1000m^a^ around junior high school without FS, CS and BS<1.

	**Parks**					
	Boys			Girls		
	β value			β value		
Body composition	Model 1	Model 2a	Model 3a	Model 1	Model 2a	Model 3b
Height z-score	0.068	0.129	-0.192	-0.541***	-0.547**	-0.546***
Weight z-score	-0.343	-0.293	-0.309*	-0.253**	-0.226**	-0.226*
BMI z-score	-0.386	-0.353	-0.278	-0.037	-0.001	-0.0004
WC z-score	-0.397*	-0.361*	-0.322	0.043	0.075	0.075
WC/Height Ratio	-0.038*	-0.036*	-0.026	0.015*	0.016*	0.018*
WC/Hip Ratio	-0.022**	-0.022**	-0.014	0.023*	0.026**	0.026**
TSF z-score	-0.654***	-0.646***	-0.549**	-0.105	-0.056	-0.055
MAMC z-score	-0.250	-0.254	-0.419	-0.022	-0.026	-0.026
	**Book and magazine rental shops**					
	Boys			Girls		
Body composition	Model 1	Model 2a	Model 3a	Model 1	Model 2a	Model 3b
Height z-score	-1.647**	-1.670*	-1.847**	-0.781	-0.870	-0.793
Weight z-score	-0.075	-0.076	0.116	-0.173	-0.237	-0.179
BMI z-score	0.561	0.571	0.841*	-0.047	-0.088	-0.050
WC z-score	0.170	0.191	0.468	0.171	0.135	0.148
WC/Height Ratio	0.048	0.050	0.079**	0.016	0.014	0.015
WC/Hip Ratio	0.033	0.035	0.057**	0.050**	0.048**	0.042*
TSF z-score	0.385	0.448*	0.816**	-0.091	-0.137	-0.067
MAMC z-score	-0.926	-0.810	-0.717	-0.573*	-0.620*	-0.570*

^a^The weighted number of food stores was calculated according to their distances from each school, with a weight of 1 for those with distances smaller than 200 m, 0.75 for those between 200–500 m, and 0.5 for those between 500 m and 1000 m.

Model 1: adjusted for father's ethnicity, mother’s education, household income, smoking, and drinking alcohol.

Model 2a: Model 1 + physical activity (moderate or heavy physical activity ≥ 30 min/day)

Model 2b: Model 1 + read during weekdays

Model 3a: Model 2 + grown beard (not yet, at first, in progress, completed)

Model 3b: Model 2 + menarche (yes, no)

*p<0.05;

**p<0.01;

***p<0.001

Here we find that, in this setting, boys are advantaged in their body composition by parks, but girls are not. But, girls’ height (indicative of growth velocity) is less. Likewise, boys’ body fatness and height are now susceptible to the reading material rental shops, dependent on puberty: and the susceptibility of girls is unchanged, irrespective of puberty.

## Discussion

### The co-operativity of facilities in the school environment in their association with students’ body composition

In this study of the environs of junior high school students, we have found those facilities which may alter energy balance to act in different directions and that the net effect depends on whether they are present and their density. More particularly, ours and others’ previous findings of the risk that fast food outlets pose to body compositional disorders (BCD)[7], may be countered by a sufficient density of physical activity facilities in the present age group. This was revealed by the modeling of BC with and without FBOs for parks whose association with less body fatness depended on the absence of FBOs, at least for boys.

### Gender differences

Gender differences in the associations of facilities with BC are evident. Whereas boys appear to benefit in their BC by the availability of fitness centers as well as gymnasia and sports stadiums, girls only benefit from gymnasia and sports stadiums. For neither boys nor girls, do other activity centers, which comprise ping-pong, basketball, dancing, music, debating, or holding meetings, have any association with BC. The association with park density was unexpectedly adverse for girls’ BC and persistent through highly adjusted models, even when FBOs were excluded, and irrespective of puberty, although the fat distributions changed somewhat with these different models.

The adverse relationships for girls BC with both park density and RMRS may be related. It is known that much of the reading material rented by girls is in the form of fiction as novels and comic books, and of magazines, with an emphasis on story-telling and relationships and on personal care. Some of this is in the genre of romance comics[38, 39] as known in Western youth culture, but which, in Taiwan may take the form of “Yaoi” where adolescent girls create and read male homosexual comic books[39]. Partly for these reasons, Taiwanese girls are the greater users of these shops than boys as well[39, 40]. It is possible and plausible that this reading material plays a role in forming body image and even BC. The counterpart is seen in aspiring sports people and in eating disorders with varying degrees of mis-match between body image and composition[41]. Parks and the RMRS may serve a similar function if girls use them to read or share their experiences with others in these settings. In this event, unlike boys, they may not increase, but rather decrease their energy expenditure through park availability. This may be compounded if girls actually avoid parks on account of personal safety. There is some evidence that personal security is a factor which contributes to BC[42].

On the other hand, we find that the beneficial association of park availability to boys is evident once FBOs are absent (see above), although the fat distribution is puberty dependent in this setting.

### The question of height

Following rapid linear growth in the first 2 years of life, the onset of the growth spurt coincides with the onset of puberty; the earlier the onset the less the magnitude of the growth spurt, although not correlated with final height[43]. But since almost 20% of final height is contributed by the pubertal growth spurt, this is a period of relative vulnerability to factors affecting stature[43]. We found that, for *girls*, the greater the availability of reading rental shops, the shorter they were, when adjusted for puberty, which suggests that non-pubertal factors (possibly what the shops provided) were affecting growth at this age. However, this was not evident when considered in those school environs with no FBOs. Perhaps the FBOs were permissive in the manifestation of the reading rental shop and height association. Similarly, for the girls in school neighborhoods without FBOs, park accessibility increased the likelihood of shortness and of abdominal fatness, irrespective of puberty. We have previously found that elementary school girls exposed to more fast food outlets are more likely to be taller and discussed how this may be relevant to the increased risk of breast cancer in pre-menopausal Taiwanese women on account of rapid growth[7]. Thus, it does not follow that the taller the better even though stunting can occur as a result of food deprivation and recurrent infection and even be adaptive to these circumstances[44–47]. Factors which are themselves not health adverse, but limit linear growth, as might be the case with reading and sedentary use of parks for social activity would not be reason to view shortness as unhealthy.

*In the case of boys*, where there were no FBOs, reading rental shop availability was associated with greater TSF thickness and also shortness irrespective of puberty; but while more park accessibility was not associated with height, it was with less fatness, with some dependence on puberty for its abdominal distribution.

In summary, reading rental shops encourage shortness in girls and boys, dependent on whether there are FBOs, and, for girls, in relation to puberty. Access to parks, probably through encouragement of physical play, reduce the risk of obesity in boys, but not in girls. Parks also have predisposition towards shortness in girls without regard to puberty, but not in boys.

### The relative importance of physical activity, sedentary and other recreational facilities

The evaluation of the relevance of school environs facilities for energy balance and BC requires their constellation to be integrated in some way and related to personal behaviors. In the present study, we have used mapping, sub-analysis and adjustment to address this need.

As far as body compositional health is concerned, only those facilities that support physical activity (fitness centers, gymnasia and sports stadiums), depending on gender, and parks (for boys where there are no FBOs), play a beneficial role. The more sedentary activities (accruing from reading material rental shops-RMRS and internet cafes) are associated with less favorable BC, for girls in all settings and, for boys undergoing puberty where there are no FBOs. Therefore, it would be expected that, once sedentary opportunities increase, the availability of physical activity facilities becomes more critical for BC health. While we did not observe an explicit association between FBOs and BC, as previously reported for elementary school environs in Taiwan[7], this was evident by the findings in their absence, which showed parks to be of BC benefit to boys if they were not present. Further sensitivity analysis did not allow a distinction to be made between the various FBOs (data not shown).

Also relevant to the potential role of FBOs in student BC is their *dietary information*. On average, across Taiwan, the students bought fast foods less than once in 2 weeks, whereas they consumed sugary beverages 5 times per week, milk almost 4 times per week and total dairy almost 6 times per week; adjustments for these or a score of dietary quality (YHEI-TW) did not explain any of the models developed to predict BC by setting. This is not to say that dietary factors are unimportant in these students BC. Given the 10-fold greater use of sugary beverages than fast foods, we would expect this to be a potential risk factor for body compositional disorders. This would be in accord with the current concern about excessive consumption of liquid energy, without the constraint of food structure on appetite, in the growing epidemic of child obesity[48].

### Puberty and the stages of physiological development in susceptibility to the school environment

The recognition of factors affecting BC during puberty is complicated by the need to distinguish physiological growth and development from its pathology and also to take account of secular trends in the onset of puberty towards younger ages[1, 24–26]. There are probably bidirectional effects of energy imbalance and earlier reproductive maturity, further compounded by associated social and behavioral immaturity[19]. Nevertheless, this is a vulnerable period for the development of BC disorders and one which merits attention to these children in their school environs, as elsewhere. We have, therefore, taken relatively unequivocal markers for the onset of puberty, the first of 4 stages in boys based on facial hair change, and one in girls, the menarche. We have adjusted our models accordingly.

We have found that puberty alters the associations between the school environs and BC and it, in turn, may be altered as part of an overall ecological disorder[49]. Girls but not boys were less often underweight after than before puberty. This may be corrective and attributable to a relative decrease in growth velocity, to a more appropriate body image at this stage of development, to change in personal behaviors to do with eating and physical activity or to other ecological factors. Inevitably, any ecological disorder will include ones to do with relationships and emotional development, themselves intimately linked to growth and development[19, 50, 51]. In contemporary medical practice, the underlying basis of evolving health patterns is increasingly understood to be ecological. This is to say that people are an integral part of their environment, especially through sensory, microbiological and endocrinological pathways[49]. Each of these conduits is now known to be pathogenetically linked to growth, development and body compositional disorders. This has extensive implications for present and future health[25–27].

## Limitations

Facilities indicative of opportunities for physical and sedentary activities have been mapped for their associations with BC, but these will not be a comprehensive set of environmental determinants of BC. However, they do show that there are synergies which may result in more or less healthful BC. This underscores the need for town and community planning to be attentive to a wide range of environmental factors in the health of schoolchildren, especially at or about puberty.

Facility availability does not necessarily represent usage- we have self-reported physical activity for which we have made adjustments in the models and which does not account for BC patterns in the different settings. Nevertheless, the reports may have had to do with activity undertaken outside the study environs.

Again, the focus on BC should not be taken as an isolated measure of student health. Thus, even though a facility may not be conducive to a healthy BC, it may provide other health benefits. A pertinent example from our work is the apparently disadvantageous associations in girls between settings with more available parks and RMRS. If these are a source of relationship-building, this may contribute to overall health[52].

## Implications

Mapping of facilities in school environs which have to do with energy balance and body composition is increasingly feasible. The most plausibly relevant are food and beverage outlets and ones which provide for physical activity, purposefully built like fitness centers and gymnasia or recreational spaces like parks and gardens. Some facilities will provide for potentially healthful activities, not necessarily reflected in body composition, like meeting places, internet cafes and libraries or other providers of reading material. Students may be affected by this environment during puberty in ways different to earlier life and later adolescence. We have shown for a representative sample of junior high school students and their school environs that the associations with sedentary activities and recreational spaces food and beverage outlets with body composition are dependent on the presence or absence of food and beverage outlets, but with beverage outlets having the greater potential risk. The accessibility of reading material providers is associated with a strikingly adverse body composition in girls with greater total and abdominal fat, irrespective of puberty and even the presence of food and beverage outlets. On the other hand, boys’ body composition is more dependent on whether there are food and beverage shops and on puberty than is the case for girls.

The findings indicate that healthy school environments require attention to a composite of facilities to which students may have access. Girls are at risk of body compositional disorders from sedentary activities in ways which may be socio-behaviorally driven and that could be mediated by alterations in energy throughput, but might also be other energy-regulatory or metabolic pathways; there are, for example, recognized cortico-hypothalamic- neuro-endocrine pathways linking behavior to intra-abdominal omental fat metabolism[53–55]. Where the age group is pubertal, the vulnerability of boys to the aggregate environment is of added concern. To speak of the aggregate environment may be unusual, but it is very much the nub of what we have found. Namely, that several heretofore unconnected facilities in the school environment may be co-operative in their net effect on child health. The documentation of these probable collective environmental hazards to the body compositional health of young adolescent students in conjunction with regular health checks should allow risk mitigation.

## Conclusions

Physical activity facilities in school environments favor healthier adolescent body composition, but differently for boys and girls (Fig 2). They act conjointly with facilities that provide sedentary activities. Food and beverage outlet absence increases the likelihood of healthier body composition for boys. The net association of facilities for physical and sedentary activity around junior high schools on body composition is dependent on stage of puberty.
